# Silencing long non‐coding RNA DLX6‐AS1 or restoring microRNA‐193b‐3p enhances thyroid carcinoma cell autophagy and apoptosis via depressing HOXA1

**DOI:** 10.1111/jcmm.16868

**Published:** 2021-09-12

**Authors:** Ling Feng, Ru Wang, Yifan Wang, Xixi Shen, Qian Shi, Meng Lian, Hongzhi Ma, Jugao Fang

**Affiliations:** ^1^ Department of Otorhinolaryngology Head and Neck Surgery Beijing Tongren Hospital Capital Medical University Beijing China; ^2^ Key Laboratory of Otorhinolaryngology Head and Neck Surgery Ministry of Education Beijing China

**Keywords:** autophagy, HOXA1, long non‐coding RNA DLX6‐AS1, metastasis, microRNA‐193b‐3p, thyroid carcinoma

## Abstract

Long non‐coding RNA DLX6 antisense RNA 1 (DLX6‐AS1) lists a critical position in thyroid carcinoma (TC) development. However, the overall comprehension about DLX6‐AS1, microRNA (miR)‐193b‐3p and homeobox A1 (HOXA1) in TC is not thoroughly enough. Concerning to this, this work is pivoted on DLX6‐AS1/miR‐193b‐3p/HOXA1 axis in TC cell growth and autophagy. TC tissues and adjacent normal thyroid tissues were collected, in which expression of DLX6‐AS1, miR‐193b‐3p and HOXA1 was tested, together with their interactions. TC cells were transfected with DLX6‐AS1/miR‐193b‐3p‐related oligonucleotides or plasmids to test cell growth and autophagy. Tumorigenesis in nude mice was observed. DLX6‐AS1 and HOXA1 were up‐regulated, and miR‐193b‐3p was down‐regulated in TC. Depleted DLX6‐AS1 or restored miR‐193b‐3p disturbed cell growth and promoted autophagy. DLX6‐AS1 targeted miR‐193b‐3p and positively regulated HOXA1. miR‐193b‐3p inhibition mitigated the impaired tumorigenesis induced by down‐regulated DLX6‐AS1. Tumorigenesis in nude mice was consistent with that in cells. It is clear that DLX6‐AS1 depletion hinders TC cell growth and promotes autophagy via up‐regulating miR‐193b‐3p and down‐regulating HOXA1.

## 1 INTRODUCTION

Thyroid carcinoma (TC), as the most prevalent endocrine malignancy, is mainly originated from thyroid follicular epithelial cells, encouraging the rise of papillary, follicular and poorly differentiated or anaplastic entities whereas about 5% TC from parathyroid follicular thyroid cells.^1^ Anaplastic thyroid carcinoma (ATC) is rare but extremely aggressive with a high mortality rate while differentiated TC is relatively common and indolent.^2^ Various therapies such as surgery, radiotherapy and chemotherapy have been implied in the use of TC, but with limitations in drug resistance, postoperative complications and side effects or unmet efficacy.^3^ Facing to the obstacles in treating TC, the requirement for therapeutic agents targeting TC positions a priority.

The co‐expression network of long non‐coding RNAs (lncRNAs) and microRNAs (miRNAs) has been extensively discussed in TC. Exactly, it is recorded that miR‐146b‐5p negatively regulates lncRNA MALAT1 to function in papillary TC (PTC) cell invasion and proliferation.^4^ Also, by co‐working with miR‐206 and miR‐599, lncRNA NEAT1 enables itself to impede ATC cell migration and invasion under hypoxic conditions.^5^ Independently, an observational study has elucidated that lncRNA DLX6 antisense RNA 1 (DLX6‐AS1) is repressive for TC cell migration and invasion.^6^ Surprisingly, miR‐193b‐3p is investigated to tie up with PTC invasiveness.^7^ However, the integrated performance of DLX6‐AS1 and targeted miR‐193b‐3p in TC has seldom been navigated. Homeobox A (HOXA) group has been suggested to take part in tumorigenesis of follicular TC, such as HOXA9.^8^ Recently, HOXA9 has been discovered to connect with invasion and migration in PTC.^9^ Specifically, it has been demonstrated that there is a significant discrepancy in methylation status of HOXA1 between benign thyroid lesions and PTC of formalin‐fixed, paraffin‐embedded tissues.^10^ Briefly, how DLX6‐AS1 and miR‐193b‐3p cooperate in the process of TC is decoded in this work from the perspective of HOXA1.

## 2 MATERIALS AND METHODS

### 2.1 Ethics statement

This study was approved by the Ethics Committee of Beijing Tongren Hospital, Capital Medical University, and the written informed consent of each participant was obtained. All animal plans were approved by the Animal Ethics Committee of Beijing Tongren Hospital, Capital Medical University.

### 2.2 Clinical specimen collection

Patients (*n* = 108) who were diagnosed with TC in Beijing Tongren Hospital, Capital Medical University, during 2017–2019 were recruited, whose cancer tissues were collected while the adjacent normal thyroid tissues were taken as controls. None of patients had received treatment (including radiotherapy and chemotherapy) before operation.

### 2.3 Cell culture

Human TC cell lines K1, BCPAP, IHH4 and TPC1 and human normal thyroid epithelial cell line Nthyori3‐1 were provided by ATCC (VA, USA) and Shanghai Institute of Biochemistry and Cell Biology, Chinese Academy of Sciences (Shanghai, China). K‐1, BCPAP, TPC1 and IHH4 cells were cultured in Dulbecco's modified Eagle's medium (DMEM, Invitrogen, CA, USA) while Nthyori3‐1 cells in Roswell Park Memorial Institute‐1640 medium (Invitrogen). The medium was supplemented with 10% foetal bovine serum (FBS, Gibco, CA, USA) and 1% penicillin/streptomycin.^11^

### 2.4 Cell transfection

Incubated in DMEM on 6‐well plates at 2 × 10^6^ cells/well, K1 cells with 50% confluence were transfected by Lipofectamine 2000 (Invitrogen). The medium was replaced 6 h later. At 48 h post‐culture, cells were collected for proceeding experiments.^12^

K1 cells were transfected with DLX6‐AS1 irrelevant plasmids, sh‐DLX6‐AS1 plasmids, overexpression (oe)‐DLX6‐AS1 plasmids, miR‐193b‐3p irrelevant sequences, miR‐193b‐3p‐mimic, miR‐193b‐3p‐inhibitor, or sh‐DLX6‐AS1 and miR‐193b‐3p‐inhibitor.

### 2.5 Reverse transcription quantitative polymerase chain reaction (RT‐qPCR)

Total RNA from tissues and cells was obtained by Trizol (Invitrogen), and the concentration and purity of RNA were evaluated by a Nanodrop 2000 spectrophotometer (Thermo Fisher Scientific, MA, USA). Complementary DNA (cDNA) was obtained by reverse transcription of RNA on the Prime‐Script RT reagent Kit (Takara, Japan) and the One Step Prime‐Script miRNA cDNA Synthesis Kit (Takara, Japan). SYBR Premix Ex Taq II (Life Tech, CA, USA) were utilized for qPCR. The primers are listed in Table 1. Glyceraldehyde‐3‐phosphate dehydrogenase (GAPDH) and U6 were considered as loading controls for genes, whose expression levels were calculated by 2^−ΔΔCt^ method.^13^

**TABLE 1:** Primer sequence

Primer sequences	Forward (5′→3′)	Reverse (5′→3′)
DLX6‐AS1	AGTTTCTCTCTAGATTGCCTT	ATTGACATGTTAGTGCCCTT
miR‐193b‐3p	GTTAACTGGCCCTCAAAGTC	GTTGGCTCTGGTGCAGGGTCCGAGGTATTCGCACCAGAGCCAACAGCGG
HOXA1	AGTTGGAGAGTACGGCTACCTG	TGCAGGGATGCAGCGATCTCCAC
U6	CTCGCTTCGGCAGCACA	AACGCTTCACGAATTTGCGT
GAPDH	GCACCGTCAAGGCTGAGAAC	TGGTGAAGACGCCAGTGGA

Note

DLX6‐AS1, long non‐coding RNA DLX6 antisense RNA 1; miR‐193b‐3p, microRNA‐193b‐3p; HOXA1, homeobox A1; GAPDH, glyceraldehyde‐3‐phosphate dehydrogenase.

### 2.6 Western blot assay

Pre‐lysed in radio‐immunoprecipitation assay cell lysis buffer (Gibco), proteins from cells and tissues were extracted by centrifugation and detected by bicinchoninic acid kit (Sangon Biotech Co., Ltd., Shanghai, China). Successively separated on sodium dodecyl sulphate‐polyacrylamide gel electrophoresis and transferred to polyvinylidene fluoride membranes, the protein samples were blocked with 5% skim milk, reacted with primary antibodies HOXA1 (1:1000, ab230513, Abcam, Cambridge, UK), LC3‐II and LC3‐I (1:1000, ABC929, Sigma‐Aldrich, CA, USA) and GAPDH (1:2500, ab9485, Abcam). Next, the protein samples were incubated with secondary antibody (1:1000, ab150077, Abcam) and developed by enhanced chemiluminescence (Millipore, MA, USA) to detect gene protein expression.^14^

### 2.7 Cell counting kit (CCK)‐8 assay

Cell suspension (100 μl) was seeded into 96‐well plates at 0.5 × 10^4^ cells/well and cultured to adherence to the wall. Then, the cells were added with 10 μl CCK‐8 solution (Yeasen, Shanghai, China) after 24, 48 and 72 h, respectively. Cells were cultured in 95% humidity for 1 h to read absorbance values at 450 nm on a microplate reader (Bio‐Rad, Hercules, CA, USA).^15^

### 2.8 Colony formation assay

K1 cells which had been transfected were detached with trypsin (Solarbio, Beijing, China) and cultured for 21 days in 10% FBS‐DMEM on 6‐well plates (Corning, N.Y., USA) at 200 cells/well. Colonies were fixed with methanol and stained with 0.5% crystal violet solution (Sigma‐Aldrich). The stained colonies were counted and photographed.

### 2.9 Flow cytometry

Annexin V‐fluorescein isothiocyanate (FITC) Apoptosis Detection Kit (556547, BD Biosciences, NJ, USA) was employed to evaluate K1 cell apoptosis. Trypsinized K1 cells were resuspended in 1× binding buffer, incubated with propidium iodide and FITC‐Annexin V and mixed with 1× binding buffer. Finally, K1 cells were detected by a flow cytometer (BD, San Jose, CA, USA).

### 2.10 Transwell assay

Cell invasion and migration were analysed by Transwell chamber (Corning) with or without coated Matrigel (BD Biosciences). K1 cells (2 × 10^4^ cells) were resuspended in 200 μl serum‐free medium and seeded into the upper chamber. The lower chamber was filled with 500 μl 10% FBS‐DMEM. Incubated for 24 h, K1 cells on the upper chamber were wiped with a cotton swab. The invading or migrating cells on the surface of the lower chamber of the filter were fixed with 4% paraformaldehyde, stained with 0.1% crystal violet solution, photographed and counted in five random fields.^16^

### 2.11 RNA pull‐down assay

K1 cell which had transfected was rinsed with PBS and incubated in a specific lysate (Ambion, Austin, Texas, USA). The lysate was mixed with M‐280 streptavidin magnesium beads (S3762, St. Louis, USA) pre‐coated with RNase‐free BSA and yeast tRNA (TRNABAK‐RO, St. Louis, Missouri, USA). After that, the sample was rinsed twice with pre‐chilled lysis buffer, 3 times with low salt buffer and once with high salt buffer. RNA was purified by Trizol, and miR‐193b‐3p expression was tested by RT‐qPCR.^17^

### 2.12 Dual‐luciferase reporter gene assay

Predicted binding sites of DLX6‐AS1, miR‐193b‐3p and HOXA1 were analysed by RNA22. Targeting relationship of DLX6‐AS1 and miR‐193b‐3p, and miR‐193b‐3p and HOXA1 was verified by dual‐luciferase reporter gene assay. The target sequence and the mutant sequence were designed and constructed separately. The target sequence and the mutant sequence were introduced into pMIR‐reporter (Beijing Huayueyang Biotechnology Co., Ltd., Beijing, China) by the endonuclease sites SpeI and HindIII. Wild‐type (WT) or mutant type (MUT) plasmid was co‐transfected into K1 cells with miR‐193b‐3p mimic or miR‐193b‐3p‐NC, respectively. After 48 h, K1 cells were lysed to examine luciferase activity by dual‐luciferase assay system (Promega, Madison, WI, USA) and GloMax20/20 Luminometer fluorescence detector (Promega). Relative luciferase activity = firefly/Renilla luciferase activity.^18^

### 2.13 Tumour xenografts in nude mice

Nude mice (4–6 weeks old, female) were originated from HFK (Beijing, China) and raised under pathogen‐free conditions. Stably transfected K1 cells (2 × 10^6^ cells) were injected subcutaneously in nude mice. Xenografted tumours were measured every 5 days: volume = (*a* × *b* ^2^)/2 (*a* was the longest diameter and *b* was the shortest diameter of the xenografted tumour). Mice were euthanized 25 days later to obtain the tumours, which were photographed and weighed.

### 2.14 Transferase‐mediated deoxyuridine triphosphate‐biotin nick end‐labelling (TUNEL) staining

Tumour tissues were fixed in 10% paraformaldehyde and embedded in paraffin. Then, the tissues were reacted with TUNEL mixture by following the guidance of the in situ cell apoptosis detection kit (POD, Roche Diagnostics GmbH, Mannheim, Germany). The stained slides were detected with an Olympus IX51 fluorescence microscope (Olympus), and the apoptosis rate was calculated.

### 2.15 Statistical analysis

All data were analysed by SPSS21.0 (IBM, NY, USA) statistical software. Measurement data were expressed as mean ± standard deviation. Discrepancy between two groups was assessed by *t* test while that among multiple groups by one‐way analysis of variance (ANOVA), followed by Tukey's post hoc test. Pearson analysis was applied in correlation assessment. At *p* < 0.05, statistical significance was set.

## 3 RESULTS

### 3.1 DLX6‐AS1 is up‐regulated and miR‐193b‐3p is down‐regulated in TC

**FIGURE 1:**
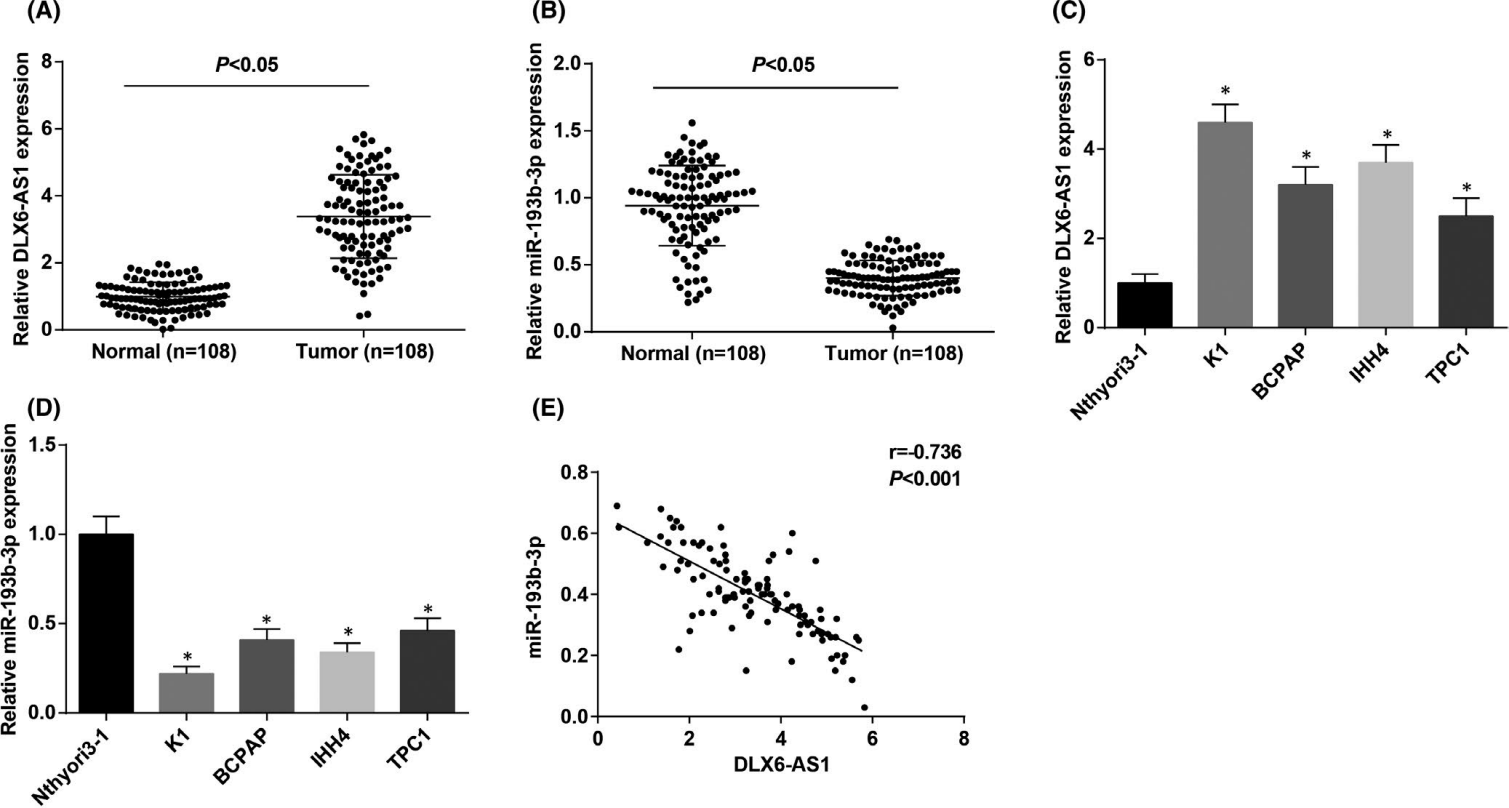
DLX6‐AS1 is up‐regulated and miR‐193b‐3p is down‐regulated in TC. (A) RT‐qPCR detected DLX6‐AS1 expression in TC cancer tissues and adjacent normal thyroid tissues (*n* = 108); (B) RT‐qPCR detected miR‐193b‐3p expression in TC cancer tissues and adjacent normal thyroid tissues (*n* = 108); (C) RT‐qPCR detected DLX6‐AS1 expression in TC cell lines and normal epithelial cells; (D) RT‐qPCR detected miR‐193b‐3p expression in TC cell lines and normal epithelial cells; (E) Pearson correlation analysis of the relation between DLX6‐AS1 and miR‐193b‐3p expression in TC tissues (*n* = 108). Measurement data were expressed as mean ± standard deviation, *N* = 3. *p < 0.05 compared with Nthyori3‐1 cells. Discrepancy between two groups was assessed by *t* test while that among multiple groups by one‐way ANOVA, followed by Tukey's post hoc test

A study has manifested overexpressed DLX6‐AS1 in TC tissues versus normal tissues.^6^ Also, DLX6‐AS1 was up‐regulated^19^ while miR‐193b‐3p was down‐regulated in other cancers.^20^, ^21^ To stratify the role of DLX6‐AS1 and miR‐193b‐3p in TC, their expression in 108 pairs of cancer tissues and adjacent normal thyroid tissues was tested by RT‐qPCR. It was displayed that cancer tissues expressed high DLX6‐AS1 and low miR‐193b‐3p expression (Figure 1A,B). In addition, DLX6‐AS1 expression was elevated and miR‐193b‐3p expression was reduced in TC cell lines versus Nthyori3‐1 cells (Figure 1C,D). Expression of miR‐193b‐3p and DLX6‐AS1 was negatively connected in TC tissues (Figure 1E).

### 3.2 Depleted DLX6‐AS1 disturbs cell progression and promotes autophagy in TC

**FIGURE 2:**
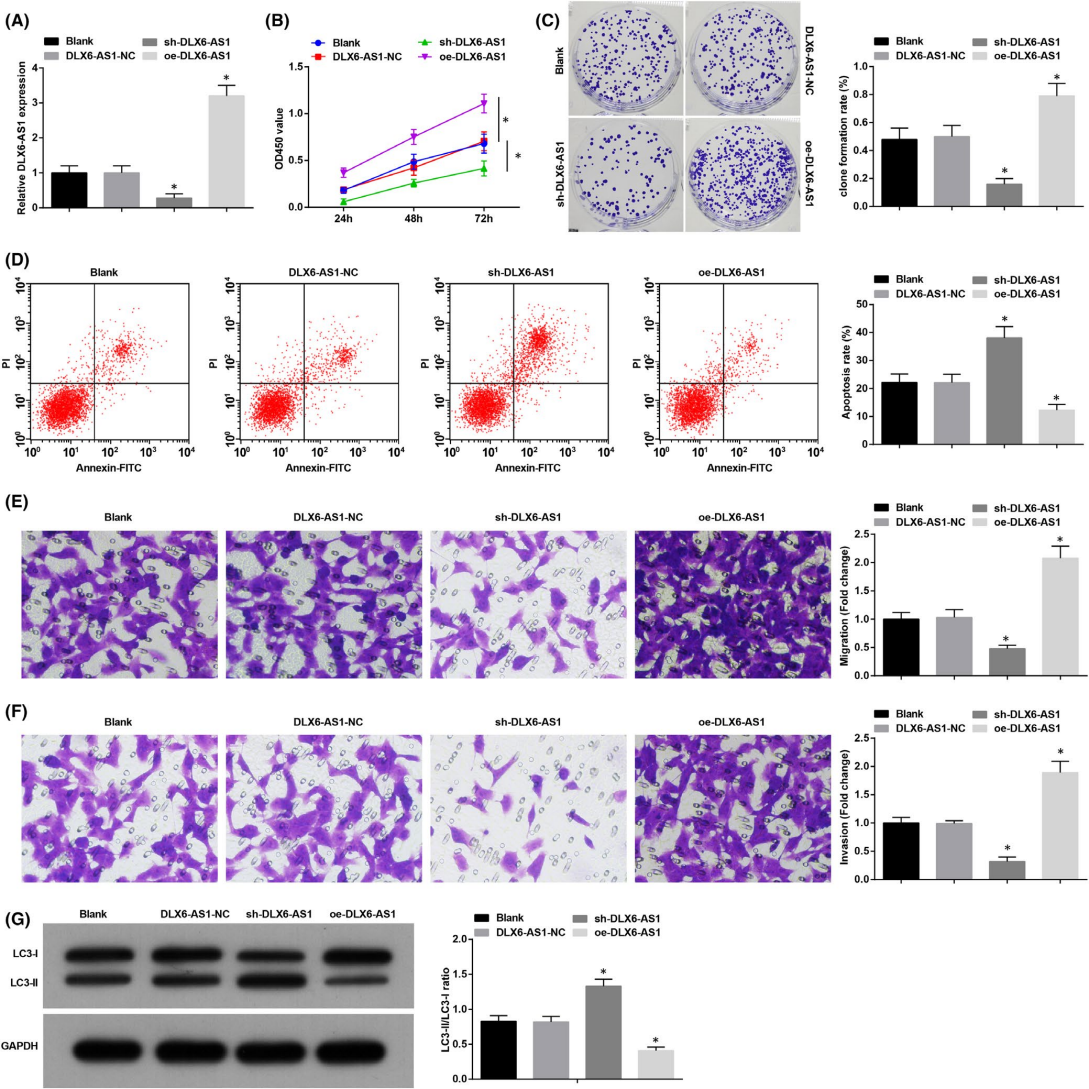
Depleted DLX6‐AS1 disturbs cell progression and promotes autophagy in TC. (A) RT‐qPCR detected DLX6‐AS1 expression in K1 cells; (B) CCK‐8 detected K1 cell proliferation; (C) Colony formation assay detected K1 cell colony‐forming ability; (D) Flow cytometry detected K1 cell apoptosis rate; (E) Transwell assay detected K1 cell migration; (F) Transwell assay detected K1 cell invasion; (G) Western blot assay detected autophagy‐related factors. Measurement data were expressed as mean ± standard deviation, *N* = 3. *p < 0.05 compared with the DLX6‐AS1‐NC group. Discrepancy among multiple groups was assessed by one‐way ANOVA, followed by Tukey's post hoc test

K1 cells were screened out to explore the functional performance of dysregulated DLX6‐AS1 in TC. RT‐qPCR confirmed that DLX6‐AS1 expression could be effectively inhibited or up‐regulated in K1 cells (Figure 2A). CCK‐8, colony formation and Transwell assays, along with flow cytometry, was in application in identifying DLX6‐AS1 regulation‐induced effects on TC cell progression. The findings concluded that depleted DLX6‐AS1 restrained K1 cell proliferation, invasion, migration and triggered apoptosis (Figure 2B‐F).

LC3 was a cytoplasmic protein (LC3‐I), which was converted into vesicular membrane‐associated protein (LC3‐II) after inducing autophagy.^22^ LC3‐II was an important marker molecule necessary for the formation of autophagosomes and selective recruitment of substrates, which increased with the increase of autophagosome membranes.^23^ Detection of LC3‐II and LC3‐I by Western blot assay manifested that LC3‐II/LC3‐I ratio increased by DLX6‐AS1 inhibition. Conversely, DLX6‐AS1 overexpression exerted the opposite effects on K1 cells (Figure 2G).

### 3.3 DLX6‐AS1 targets miR‐193b‐3p

**FIGURE 3:**
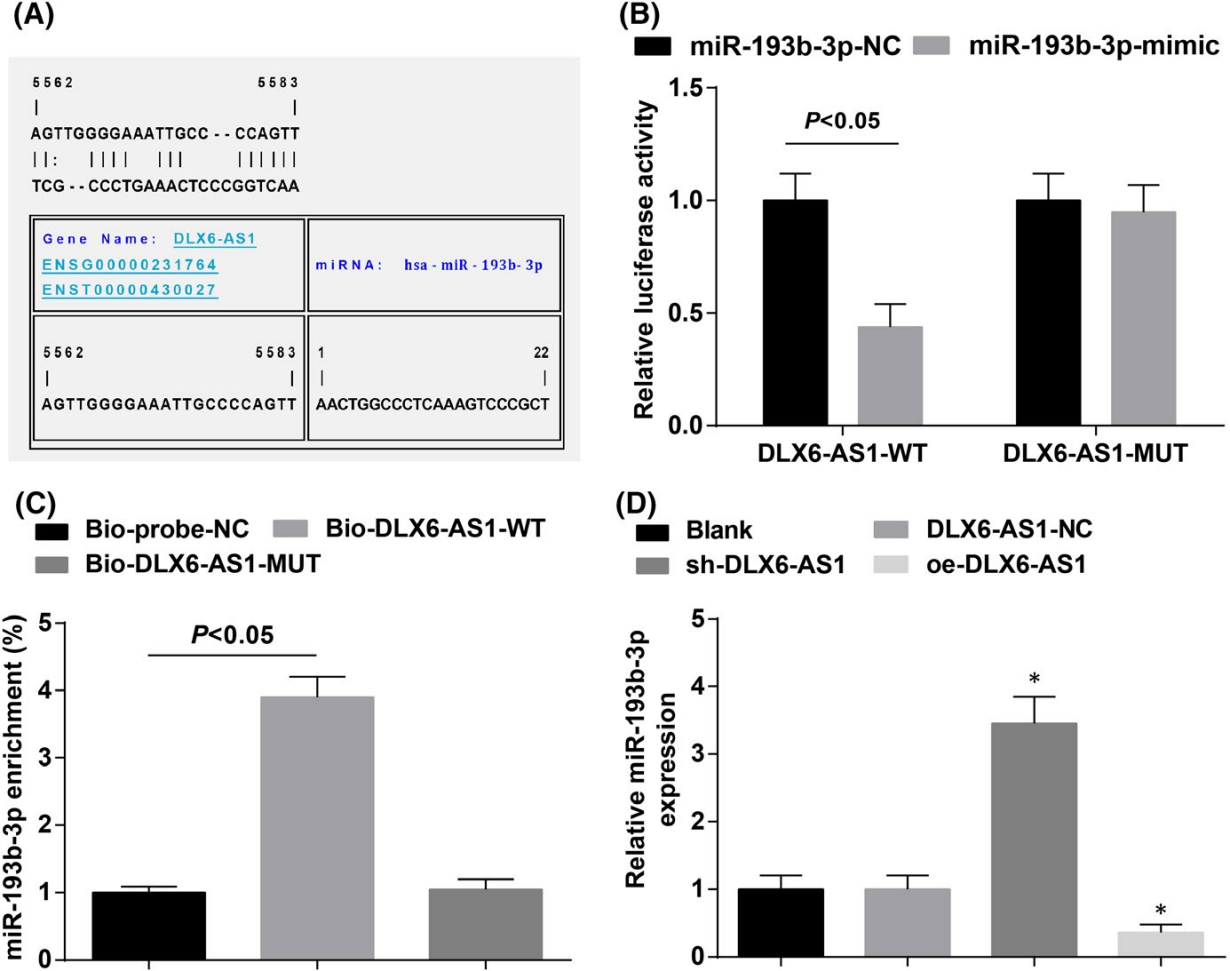
DLX6‐AS1 targets miR‐193b‐3p. (A) RNA22 website predicted the binding site of miR‐193b‐3p and DLX6‐AS1; (B) Dual‐luciferase reporter gene assay verified the targeting relationship between DLX6‐AS1 and miR‐193b‐3p; (C) RNA pull‐down assay tested the relationship between DLX6‐AS1 and miR‐193b‐3p; (D) RT‐qPCR detected miR‐193b‐3p expression in K1 cells after DLX6‐AS1 inhibition or overexpression. Measurement data were expressed as mean ± standard deviation, *N* = 3. *p < 0.05 compared with the DLX6‐AS1‐NC group. Discrepancy between two groups was assessed by *t* test while that among multiple groups by one‐way ANOVA, followed by Tukey's post hoc test

The regulatory mechanism of DLX6‐AS1 and miR‐193b‐3p was firstly tested by RNA22 to predict the binding site between the two (Figure 3A) and then by dual‐luciferase reporter gene assay to verify the binding of miR‐193b‐3p a DLX6‐AS1 (Figure 3B). It was outlined that miR‐193b‐3p‐mimic caused an impairment in fluorescence activity of DLX6‐AS1‐WT while had nothing to do with fluorescence activity of DLX6‐AS1‐MUT. RNA pull‐down assay depicted that (Figure 3C) miR‐193b‐3p was enriched in Bio‐DLX6‐AS1‐WT of K1 cells while its enrichment was not changed in Bio‐DLX6‐AS1‐MUT. RT‐qPCR demonstrated that DLX6‐AS1 depletion or restoration led to miR‐193b‐3p expression elevation or reduction, respectively (Figure 3D).

### 3.4 Restored miR‐193b‐3p disturbs cell progression and promotes autophagy in TC

**FIGURE 4:**
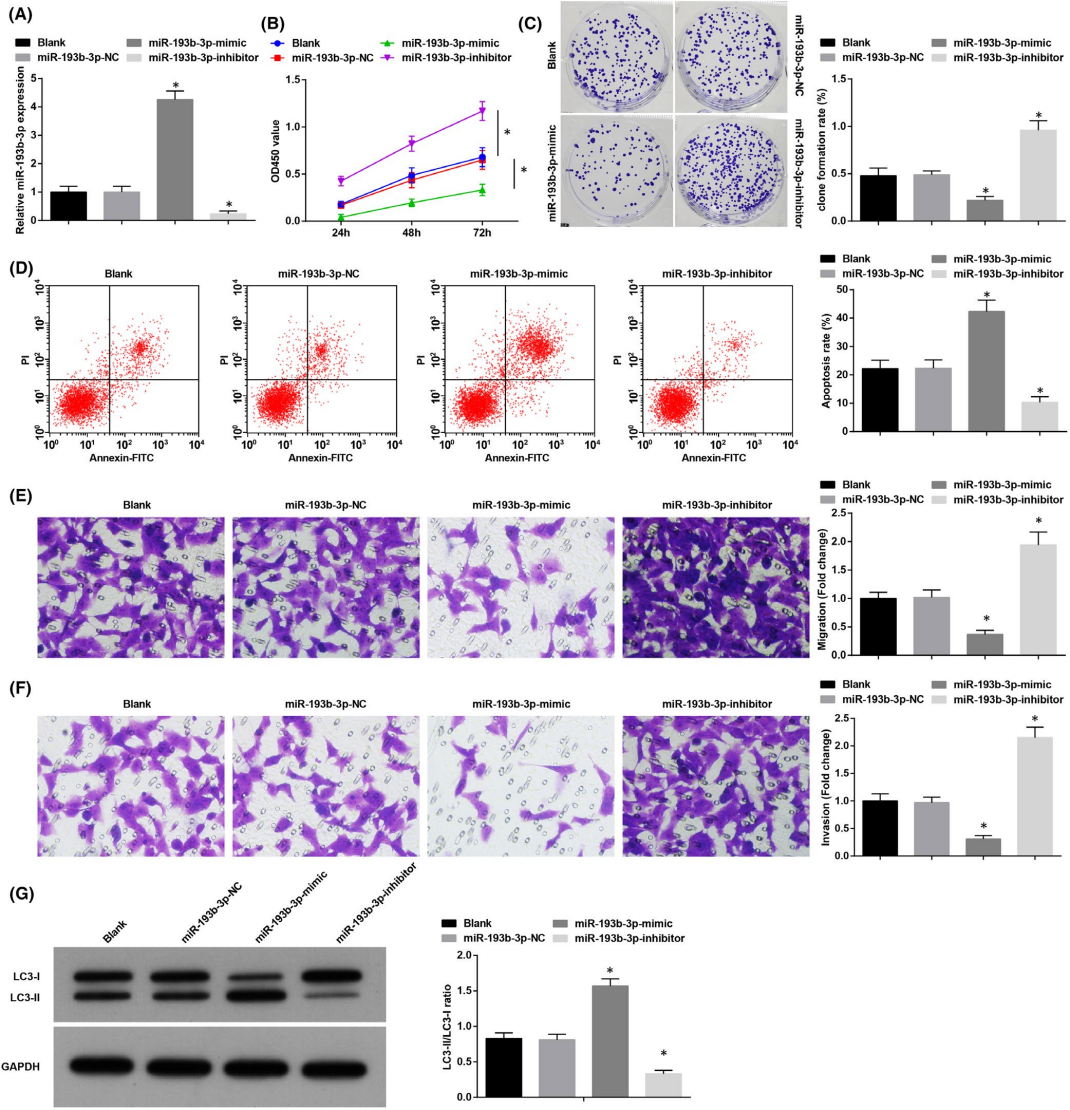
Restored miR‐193b‐3p disturbs cell progression and promotes autophagy in TC. (A) RT‐qPCR detected miR‐193b‐3p expression in K1 cells; (B) CCK‐8 detected K1 cell proliferation; (C) colony formation assay detected K1 cell colony‐forming ability; (D) flow cytometry detected K1 cell apoptosis rate; (E) Transwell assay detected K1 cell migration; (F) Transwell assay detected K1 cell invasion; (G) Western blot assay detected autophagy‐related factors. Measurement data were expressed as mean ± standard deviation, *N* = 3. *p < 0.05 compared with the miR‐193b‐3p‐NC group. Discrepancy among multiple groups was assessed by one‐way ANOVA, followed by Tukey's post hoc test

miR‐193b‐3p mimic or miR‐193b‐3p inhibitor was transferred into K1 cells to regulate miR‐193b‐3p expression (Figure 4A). Cellular analysis by various assays disclosed that miR‐193b‐3p up‐regulation blocked the way for K1 cells to proliferate, invade, migrate and form colonies, but enhanced apoptosis and autophagy. Reversely, miR‐193b‐3p down‐regulation worked out the opposite outcomes in K1 cells (Figure 4B‐G).

### 3.5 DLX6‐AS1 positively regulates HOXA1 in TC

**FIGURE 5:**
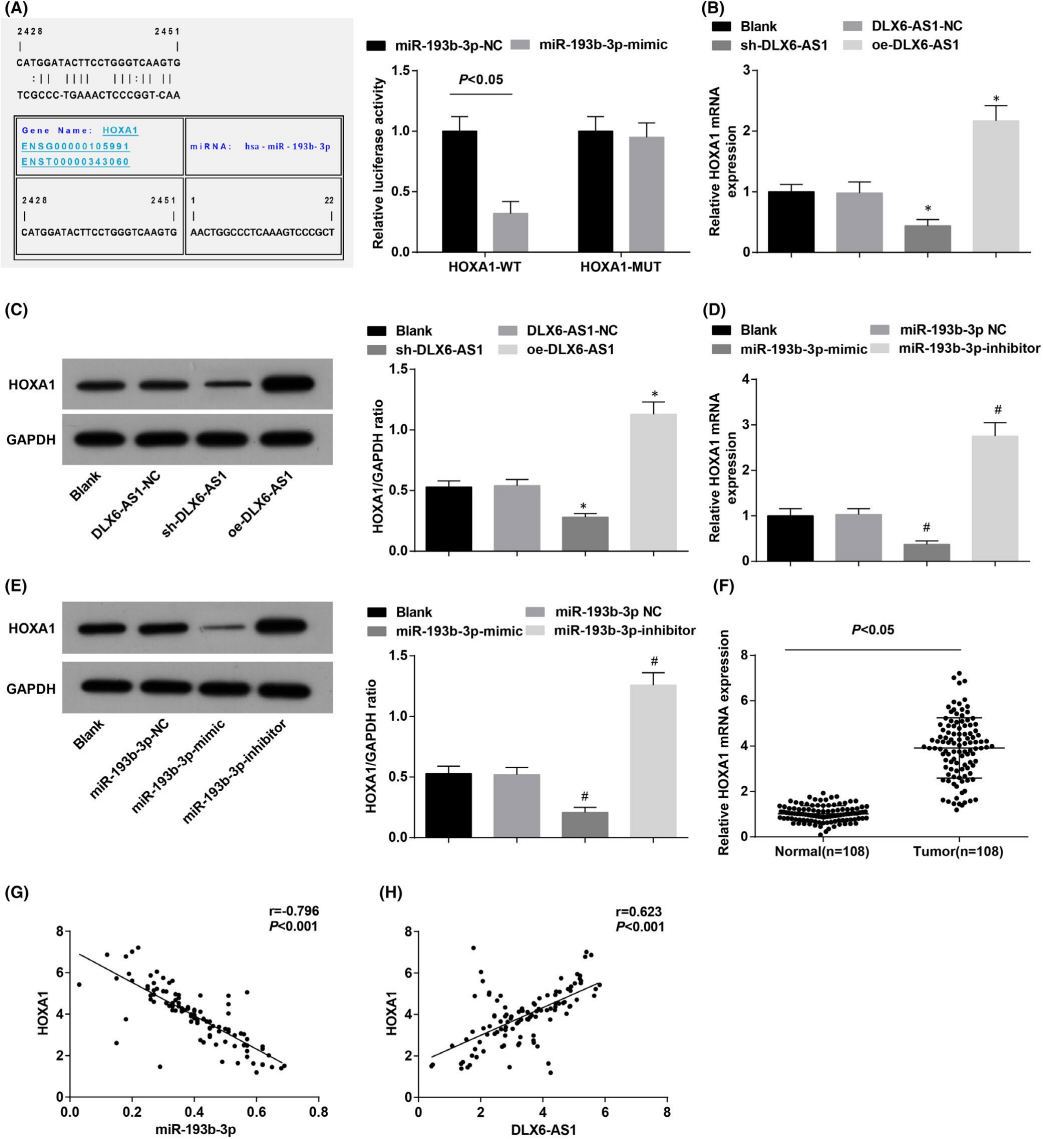
DLX6‐AS1 positively regulates HOXA1 in TC. (A) RNA22 software analysed the binding site of miR‐193b‐3p to HOXA1 and dual‐luciferase reporter gene assay analysed the binding relationship between miR‐193b‐3p and HOXA1; (B) RT‐qPCR detected HOXA1 mRNA expression in K1 cells after DLX6‐AS1 expression regulation; (C) Western blot detected HOXA1 protein expression in K1 cells after DLX6‐AS1 expression regulation; (D) RT‐qPCR detected HOXA1 mRNA expression in K1 cells after miR‐193b‐3p expression regulation; (E) Western blot detected HOXA1 protein expression in K1 cells after miR‐193b‐3p expression regulation (F) RT‐qPCR detected HOXA1 mRNA expression in TC cancer tissues and adjacent normal thyroid tissues (*n* = 108); (G) Pearson correlation analysis of the relation between miR‐193b‐3p and HOXA1 expression in TC tissues; (H) Pearson correlation analysis of the relation between HOXA1 and DLX6‐AS1 expression in TC tissues. Measurement data were expressed as mean ± standard deviation, *N* = 3. *p < 0.05 compared with the DLX6‐AS1‐NC group; #*p* < 0.05 compared with the miR‐193b‐3p‐NC group. Discrepancy between two groups was assessed by *t* test while that among multiple groups by one‐way ANOVA, followed by Tukey's post hoc test

It has been surveyed that the oncogene HOXA1 was overexpressed in tumour tissues^24^ and cells.^25^ In this work, it was implied that miR‐193b‐3p could bind to HOXA1 3′‐untranslated region and regulate HOXA1 expression in K1 cells (Figure 5A). Additionally, RT‐qPCR and Western blot assay depicted that silencing DLX6‐AS1 or restoring miR‐193b‐3p accredited to inhibited HOXA1 in K1 cells, and vice versus (Figure 5B‐E). HOXA1 was up‐regulated in TC cancer tissues (Figure 5F). miR‐193b‐3p and HOXA1 were negatively connected (Figure 5G) while DLX6‐AS1 and HOXA1 were positively connected in TC (Figure 5H).

### 3.6 miR‐193b‐3p inhibition mitigates impaired tumorigenesis induced by down‐regulated DLX6‐AS1 in TC

**FIGURE 6:**
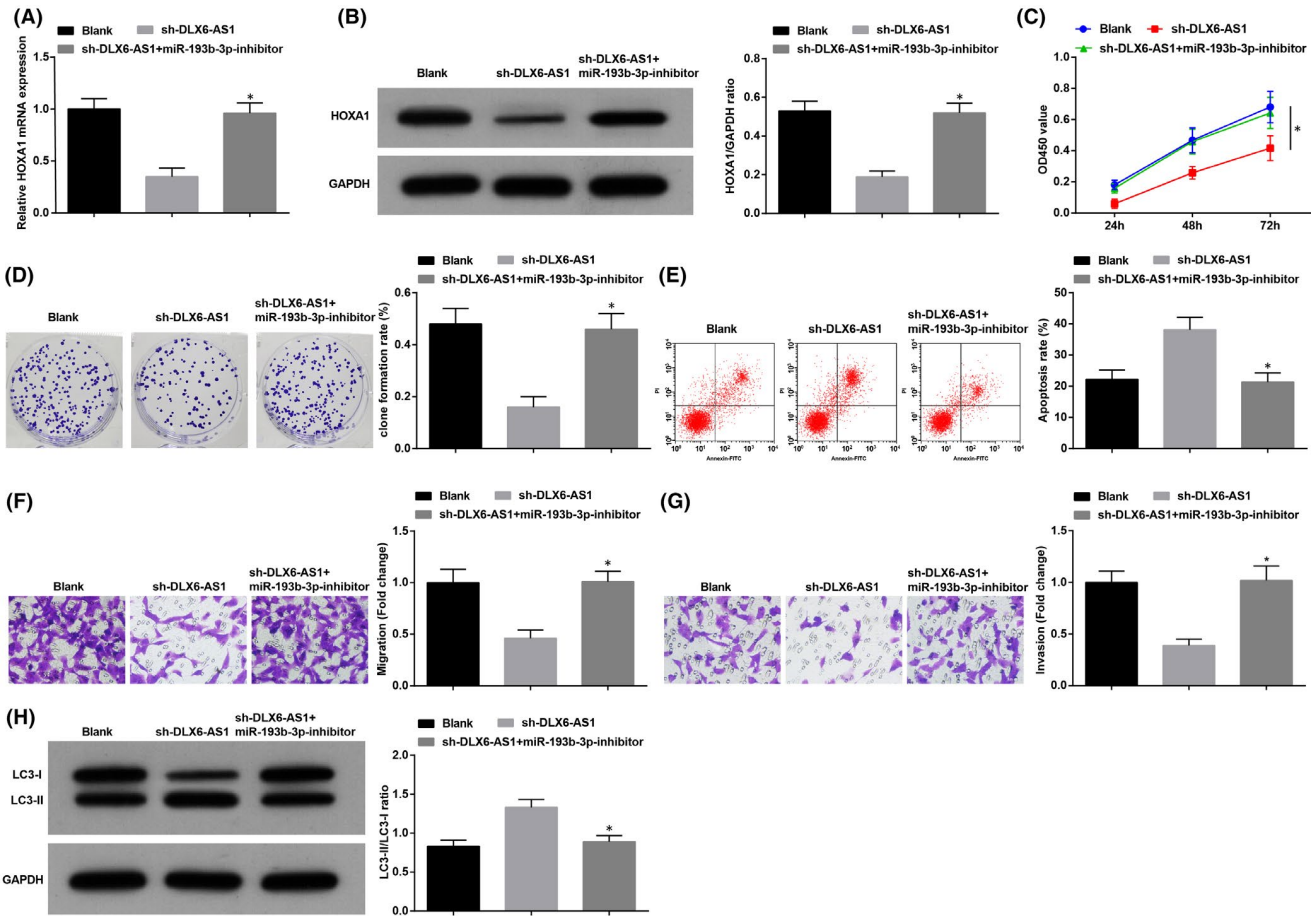
miR‐193b‐3p inhibition mitigates down‐regulated DLX6‐AS1‐induced impaired tumorigenesis of TC. (A) RT‐qPCR detected HOXA1 mRNA expression in K1 cells; (B) Western blot assay detected HOXA1 protein expression in K1 cells; (C) CCK‐8 detected K1 cell proliferation; (D) colony formation assay detected K1 cell colony‐forming ability; (E) Flow cytometry detected K1 cell apoptosis rate; (F) Transwell assay detected K1 cell migration; (G) Transwell assay detected K1 cell invasion; (H) Western blot assay detected autophagy‐related factors. Measurement data were expressed as mean ±standard deviation, *N* = 3. *p < 0.05 compared with the sh‐DLX6‐AS1 group. Discrepancy among multiple groups was assessed by one‐way ANOVA, followed by Tukey's post hoc test

To determine the carcinogenic function of miR‐193b‐3p‐mediated DLX6‐AS1 in TC, K1 cells with stably and lowly expressed DLX6‐AS1 were further transfected with miR‐193b‐3p‐inhibitor. A series of test results showed that the inhibition effect of DLX6‐AS1 on K1 cell growth and the promotion effect of autophagy were reversed by down‐regulation of miR‐193b‐3p (Figure 6C‐H).

### 3.7 Silencing DLX6‐AS1 or restoring miR‐193b‐3p impedes tumour formation in mice with TC

**FIGURE 7:**
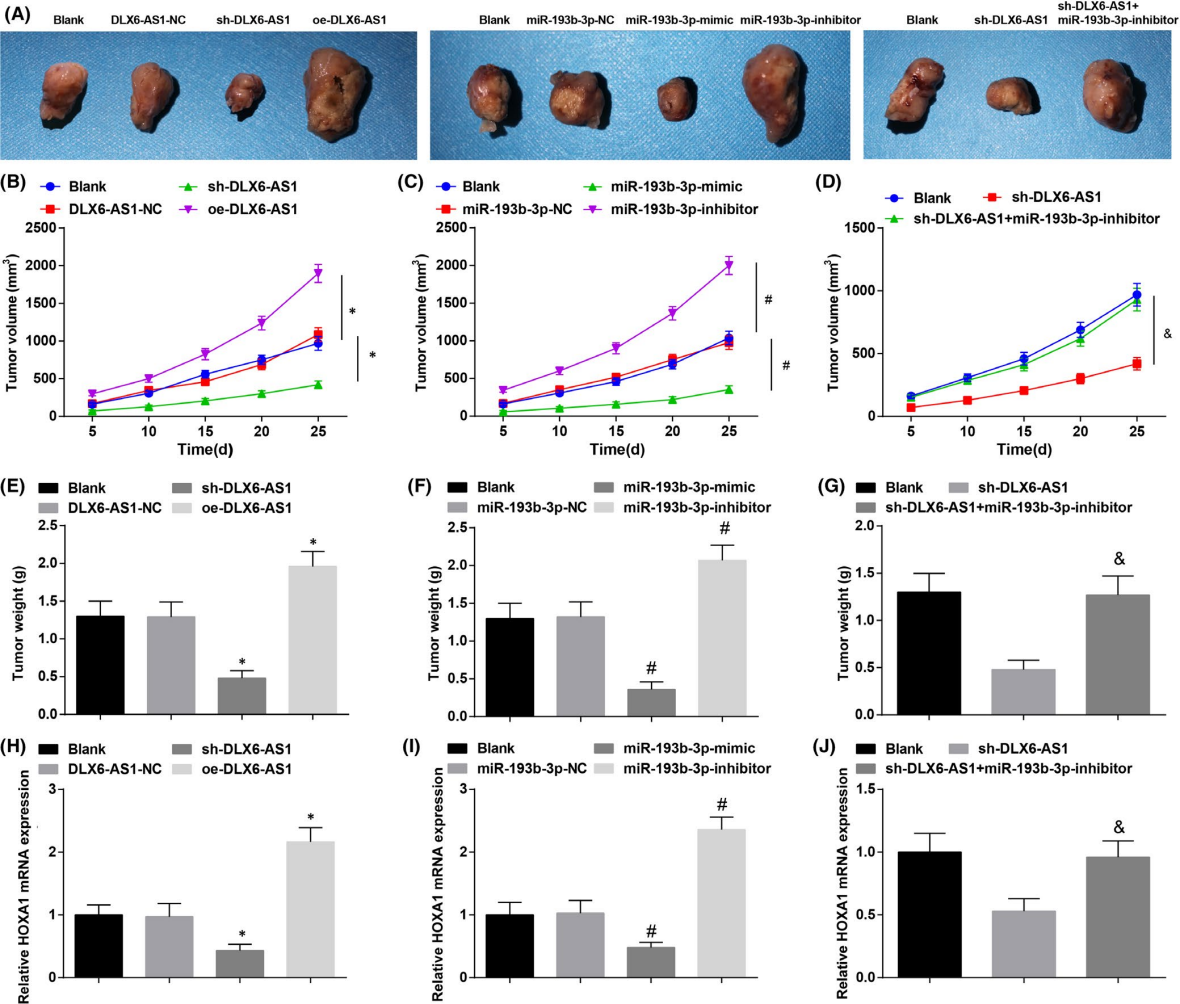
Silencing DLX6‐AS1 or restoring miR‐193b‐3p impedes tumour formation in mice with TC. (A) Representative images of xenografted tumours; (B‐D) Curves of tumour volume in nude mice; (E‐G) Tumour weight in nude mice; H‐J. HOXA1 expression in xenografted tumours; *p < 0.05 compared with the DLX6‐AS1‐NC group; #*p* < 0.05 compared with the miR‐193b‐3p‐NC group; &*p* < 0.05 compared with the sh‐DLX6‐AS1 group. Measurement data were expressed as mean ± standard deviation, *n* = 5. Discrepancy among multiple groups was assessed by one‐way ANOVA, followed by Tukey's post hoc test

To verify whether the regulatory function of DLX6‐AS1 in TC occurrence was regulated by miR‐193b‐3p/HOXA1 axis *in vivo*, K1 cells were injected subcutaneously into the dorsal side of nude mice and the tumour volume was monitored. It was clear that knocking out DLX6‐AS1 or restoring miR‐193b‐3p depressed tumour volume and weight. Overexpressing DLX6‐AS1 or inhibiting miR‐193b‐3p caused reversal results. miR‐193b‐3p restriction negated DLX6‐AS1 suppression‐induced impacts on tumours (Figure 7A‐G). Also, HOXA1 expression was measured by RT‐qPCR. The findings implied that after knockdown of DLX6‐AS1 or overexpression of miR‐193b‐3p, HOXA1 expression in xenografted tumours was inhibited, and vice versa. Moreover, inhibiting miR‐193b‐3p reversed the effect of DLX6‐AS1 knockdown on HOXA1 expression (Figure 7H‐J).

**FIGURE 8:**
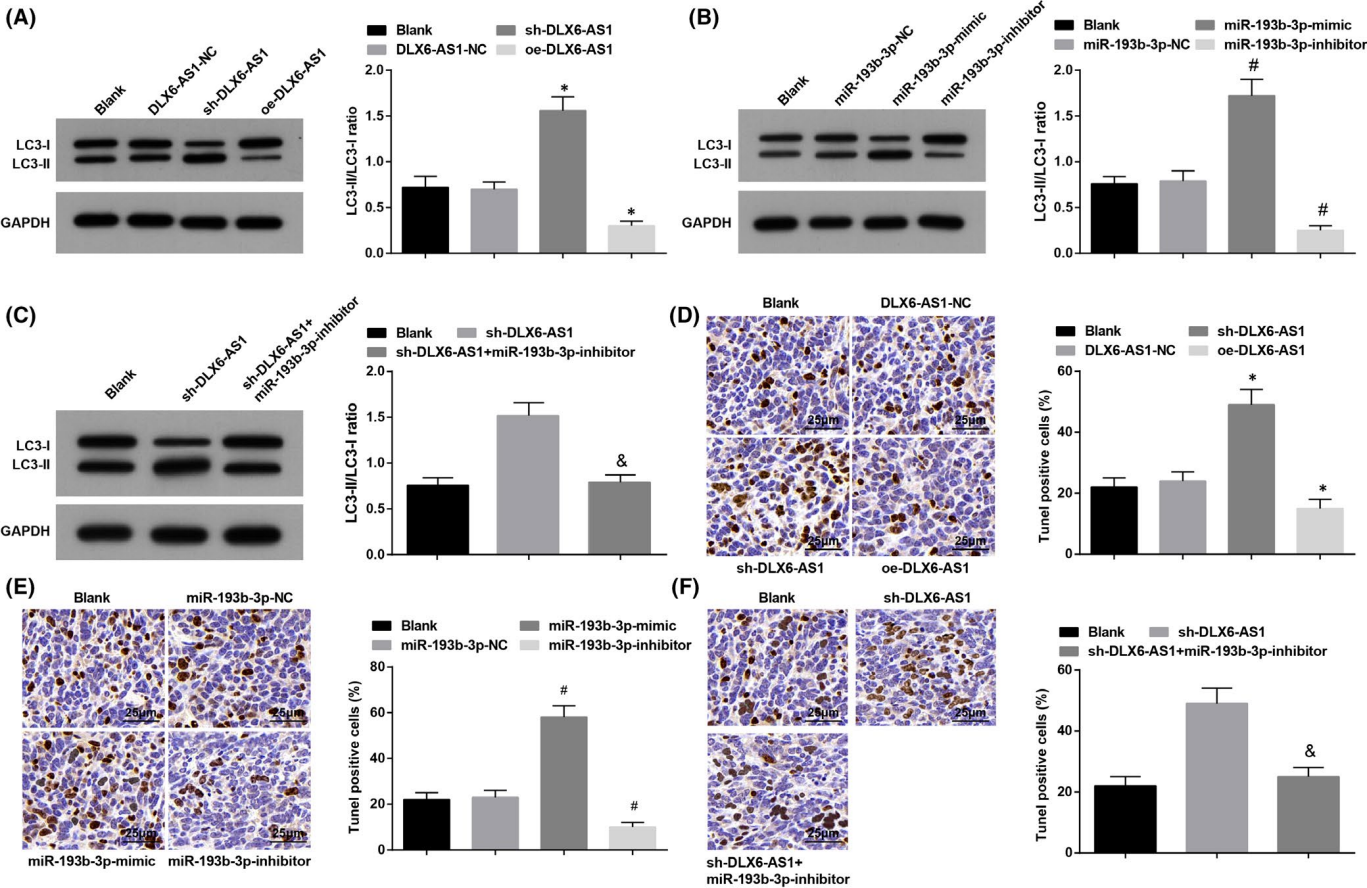
Silencing DLX6‐AS1 or restoring miR‐193b‐3p enhances autophagy and apoptosis in tumours in mice with TC. (A‐C) LC3‐II and LC3‐I protein levels in xenografted tumours; (D‐F) Apoptosis in xenografted tumours from nude mice detected using TUNEL staining (400×); *p < 0.05 compared with the DLX6‐AS1‐NC group; #*p* < 0.05 compared with the miR‐193b‐3p‐NC group; &*p* < 0.05 compared with the sh‐DLX6‐AS1 group; Measurement data were expressed as mean ± standard deviation, *n* = 5. Discrepancy among multiple groups was assessed by one‐way ANOVA, followed by Tukey's post hoc test

Autophagy and apoptosis in tumours of mice were measured by Western blot and TUNEL staining, The results reflected that after knocking down DLX6‐AS1 or up‐regulating miR‐193b‐3p, autophagy and apoptosis were enhanced, and vice versa. Also, down‐regulation of miR‐193b‐3p mitigated DLX6‐AS1 silencing‐induced importance in autophagy and apoptosis (Figure 8A‐F).

## 4 DISCUSSION

Rarely occurred TC is a heterogeneous disease, accounting for less than 1% of all tumours.^26^ LncRNAs and miRNAs are elaborated to interplay in malignant tumours, including TC. Finitely, the concrete roles of DLX6‐AS1 and miR‐193b‐3p in TC have been not downright depicted. Therein, this work has started from the glimpse of DLX6‐AS1 and miR‐193b‐3p as well as HOXA1 to summarize that DLX6‐AS1 competitively binds to miR‐193b‐3p to enhance HOXA1 expression, thereby to encourage TC cell progression and suppress autophagy (Figure S1).

Importantly, this work has delved out DLX6‐AS1 is expressed at a high level in TC. Pertaining to the functional performance DLX6‐AS1 in TC cell progression, DLX6‐AS1 regulation assays have been executed on K1 cells with the discoveries concluding that silencing DLX6‐AS1 hinders K1 cells to proliferate, invade, migrate and form colonies and supports apoptosis and autophagy (as reflected by elevated LC3‐II, LC3‐I and p62/SQSTM1 expression). Evidenced by a late research, DLX6‐AS1 expression is elevated in TC and down‐regulating it accredits to suppressed TC cell aggressive behaviours.^6^ Explored in other cancers, the inhibitory function of DLX6‐AS1 has also echoed with the discoveries in this work. For instance, in laryngeal cancer, DLX6‐AS1 expression trends towards an elevation in clinical cancer tissues and its depletion obstruct the proliferative activity of HEp‐2 and Tu‐177 cells.^27^ Also, increased DLX6‐AS1 is presented in colorectal cancer tissues and depleting DLX6‐AS1 restricts metastasis of colorectal cancer cells and arrests cell phase progression.^28^ Besides, oesophageal squamous cell carcinoma specimens express elevated DLX6‐AS1 and DLX6‐AS1 down‐regulation is attributed to depressed cell proliferation and metastasis.^29^

Crucially, the network between DLX6‐AS1 and its regulatory gene miR‐193b‐3p has been deciphered and the results imply that DLX6‐AS1 is negatively connected with and targeted to miR‐193b‐3p. Then, focused on miR‐193b‐3p, we have investigated that it is expressed at a low level in TC, and its restoration attributes to inhibited TC cell progression and reinforced autophagy. In fact, the targeting relationship between DLX6‐AS1 and miR‐193b‐3p has not been verified yet in previous researches, which requires further validation. Experimentally, a study has made it clear that miR‐193b‐3p expression is decreased in metastatic TC by comparison with non‐invasive TC tissues.^7^ Mechanistically, miR‐193b‐3p expression trends to down‐regulate in osteosarcoma and miR‐193b‐3p depletion would result in enhanced cell proliferation, reduced G0/G1 phase and impaired apoptosis.^30^ Another study also stresses out that miR‐193b‐3p expression is reduced in lung cancer and miR‐193b‐3p elevation restrains migration, invasion and colony formation activities of cells.^31^ For validation, tumorigenesis in mice is conducted to highlight that down‐regulating DLX6‐AS1 or up‐regulating miR‐193b‐3p depresses tumour formation and miR‐193b‐3p inhibition would negate DLX6‐AS1 down‐regulation‐contributed impacts on tumour weight and volume. Down‐regulating DLX6‐AS1 in gastric cancer is revealed to disturb cell viability and colony‐forming ability while stimulating apoptosis as well as restrict tumour formation in animals.^32^ Additionally, miR‐193b‐3p restoration weakens the aggressive actions of malignant cells and delays xenografted tumour growth in animal models.^33^ Anyway, DLX6‐AS1 down‐regulation and miR‐193b‐3p up‐regulation are proved to be anti‐tumour in malignant cancers, including but not limited to TC.

To proceed, the mechanism of miR‐193b‐3p/DLX6‐AS1 with HOXA1 is deeply explored to reveal a negative connection between miR‐193b‐3p and HOXA1 and the positive one between DLX6‐AS1 and HOXA1. Actually, HOXA1 expression is elevated in clinical TC tissues. Nearly no researches have depicted the regulatory interactions between HOXA1 and miR‐193b‐3p/DLX6‐AS1. HOXA1 expression is tended to elevate in nasopharyngeal carcinoma cells and up‐regulating HOXA1 leads to promoted nasopharyngeal carcinoma cell growth and proliferation.^34^ In addition, elevated HOXA1 is exhibited in type I endometrial cancer, and knocking down HOXA1 is benefit to suppressing cell proliferative, migratory and invasive capacities.^35^

Jointly, the work has delineated that DLX6‐AS1 exerts as a competing endogenous RNA for miR‐193b‐3p to target HOXA1, further to promote TC tumorigenesis, which supplies a creative option for TC treatment. Restricted to the relative small scale, the results concluded in the work should be verified in a relatively larger cohort.

## CONFLICTS OF INTEREST

The authors declare that they have no conflicts of interest.

## AUTHOR CONTRIBUTIONS

**Ling Feng:** Formal analysis (equal); Funding acquisition (equal); Resources (equal). **Ru Wang:** Investigation (equal); Methodology (equal); Project administration (equal). **Yifan Wang:** Software (equal); Writing‐original draft (equal); Writing‐review & editing (equal). **Xixi Shen:** Validation (equal); Visualization (equal); Writing‐original draft (equal). **Qian Shi:** Funding acquisition (equal); Validation (equal); Writing‐review & editing (equal). **Meng Lian:** Funding acquisition (equal); Writing‐original draft (equal). **Hongzhi Ma:** Conceptualization (equal); Funding acquisition (equal); Supervision (equal). **Jugao Fang:** Conceptualization (equal); Data curation (equal); Supervision (equal).

## Supporting information

Fig S1

## Data Availability

The data that support the findings of this study are available from the corresponding author upon reasonable request.

## References

[1] Petrulea MS , Plantinga TS , Smit JW , Georgescu CE , Netea‐Maier RT . PI3K/Akt/mTOR: a promising therapeutic target for non‐medullary thyroid carcinoma. Cancer Treat Rev. 2015;41(8):707‐713. 26138515 10.1016/j.ctrv.2015.06.005

[2] Skwiersky S , Hevroni G , Singh G , et al. Concurrent anaplastic and papillary thyroid carcinomas: a case report. Am J Med Case Rep. 2020;8(7):202‐205.32432164

[3] Yang Y , Yu W‐Y , Zhang H‐H , et al. Herbal active ingredients: an emerging potential for the prevention and treatment of papillary thyroid carcinoma. Biomed Res Int. 2020;2020:1340153. 32090065 10.1155/2020/1340153 PMC7013308

[4] Peng Y , Fang X , Yao H , Zhang Y , Shi J . MiR‐146b‐5p regulates the expression of long noncoding RNA MALAT1 and its effect on the invasion and proliferation of papillary thyroid cancer. Cancer Biother Radiopharm. 2020;36:433‐440. 32343601 10.1089/cbr.2019.3322

[5] Tan X , Weng P , Lou J , Zhang J . Knockdown of lncRNA NEAT1 suppresses hypoxia‐induced migration, invasion and glycolysis in anaplastic thyroid carcinoma cells through regulation of miR‐206 and miR‐599. Cancer Cell Int. 2020;20:132. 32336952 10.1186/s12935-020-01222-x PMC7178727

[6] Zhong ZB , Luo J‐N , Yuan Z‐N , et al. Knockdown of long noncoding RNA DLX6‐AS1 inhibits migration and invasion of thyroid cancer cells by upregulating UPF1. Eur Rev Med Pharmacol Sci. 2019;23(24):10867‐10873. 31858555 10.26355/eurrev_201912_19790

[7] Akyay OZ , Gov E , Kenar H , et al. Mapping the molecular basis and markers of papillary thyroid carcinoma progression and metastasis using global transcriptome and microRNA profiling. OMICS. 2020;24(3):148‐159. 32073999 10.1089/omi.2019.0188

[8] Jacques C , Guillotin D , Fontaine JF , et al. DNA microarray and miRNA analyses reinforce the classification of follicular thyroid tumors. J Clin Endocrinol Metab. 2013;98(5):E981‐E989. 23569218 10.1210/jc.2012-4006

[9] Jin Y , Kim HK , Lee J , et al. Transcription factor HOXA9 is linked to the calcification and invasion of papillary thyroid carcinoma. Sci Rep. 2019;9(1):6773. 31043660 10.1038/s41598-019-43207-5 PMC6494860

[10] Chang H , Shin BK , Kim A , Kim HK , Kim BH . DNA methylation analysis for the diagnosis of thyroid nodules ‐ a pilot study with reference to BRAF(V) (600E) mutation and cytopathology results. Cytopathology. 2016;27(2):122‐130. 25988212 10.1111/cyt.12248

[11] Perez‐Lachaud G , Lachaud JP . Hidden biodiversity in entomological collections: the overlooked co‐occurrence of dipteran and hymenopteran ant parasitoids in stored biological material. PLoS One. 2017;12(9):e0184614. 28926617 10.1371/journal.pone.0184614 PMC5604966

[12] Ba S , Xuan Y , Long Z‐W , Chen H‐Y , Zheng S‐S . MicroRNA‐27a promotes the proliferation and invasiveness of colon cancer cells by targeting SFRP1 through the Wnt/beta‐catenin signaling pathway. Cell Physiol Biochem. 2017;42(5):1920‐1933. 28772260 10.1159/000479610

[13] Livak KJ , Schmittgen TD . Analysis of relative gene expression data using real‐time quantitative PCR and the 2(‐Delta Delta C(T)) Method. Methods. 2001;25(4):402‐408. 11846609 10.1006/meth.2001.1262

[14] Gentile E , Ricci K , Delussi M , de Tommaso M . Motor cortex function in fibromyalgia: a pilot study involving near‐infrared spectroscopy and co‐recording of laser‐evoked potentials. Funct Neurol. 2019;34(2):107‐118. 31556391

[15] Tian F , Chen Z , Zhang Y , Jiang J , Li T . Salidroside protects LPS‐induced injury in human thyroid follicular epithelial cells by upregulation of MiR‐27a. Life Sci. 2018;213:1‐8. 30300656 10.1016/j.lfs.2018.10.006

[16] Guan H , Liang W , Xie Z , et al. Down‐regulation of miR‐144 promotes thyroid cancer cell invasion by targeting ZEB1 and ZEB2. Endocrine. 2015;48(2):566‐574. 24968735 10.1007/s12020-014-0326-7

[17] Chen S , Chen J‐Z , Zhang J‐Q , et al. Silencing of long noncoding RNA LINC00958 prevents tumor initiation of pancreatic cancer by acting as a sponge of microRNA‐330‐5p to down‐regulate PAX8. Cancer Lett. 2019;446:49‐61. 30639194 10.1016/j.canlet.2018.12.017

[18] Wang ZY , Duan Y , Wang P . SP1‐mediated upregulation of lncRNA SNHG4 functions as a ceRNA for miR‐377 to facilitate prostate cancer progression through regulation of ZIC5. J Cell Physiol. 2020;235(4):3916‐3927. 31608997 10.1002/jcp.29285 PMC13482669

[19] Liang Y , Zhang C‐D , Zhang C , Dai D‐Q . DLX6‐AS1/miR‐204‐5p/OCT1 positive feedback loop promotes tumor progression and epithelial‐mesenchymal transition in gastric cancer. Gastric Cancer. 2020;23(2):212‐227. 31463827 10.1007/s10120-019-01002-1

[20] Khordadmehr M , Shahbazi R , Sadreddini S , Baradaran B . miR‐193: a new weapon against cancer. J Cell Physiol. 2019;234(10):16861‐16872. 30779342 10.1002/jcp.28368

[21] Zhang J , Qin J , Su Y . miR‐193b‐3p possesses anti‐tumor activity in ovarian carcinoma cells by targeting p21‐activated kinase 3. Biomed Pharmacother. 2017;96:1275‐1282. 29169729 10.1016/j.biopha.2017.11.086

[22] Roldan JS , Candurra NA , Colombo MI , Delgui LR . Junin virus promotes autophagy to facilitate the virus life cycle. J Virol. 2019;93(15):1‐16. 10.1128/JVI.02307-18 PMC6639288 31118257

[23] Jiang TX , Zou JB , Zhu QQ , et al. SIP/CacyBP promotes autophagy by regulating levels of BRUCE/Apollon, which stimulates LC3‐I degradation. Proc Natl Acad Sci U S A. 2019;116(27):13404‐13413. 31213539 10.1073/pnas.1901039116 PMC6613085

[24] Li Q , Dong C , Cui J , Wang Y , Hong X . Over‐expressed lncRNA HOTAIRM1 promotes tumor growth and invasion through up‐regulating HOXA1 and sequestering G9a/EZH2/Dnmts away from the HOXA1 gene in glioblastoma multiforme. J Exp Clin Cancer Res. 2018;37(1):265. 30376874 10.1186/s13046-018-0941-x PMC6208043

[25] Yuan C , Zhu X , Han Y , et al. Elevated HOXA1 expression correlates with accelerated tumor cell proliferation and poor prognosis in gastric cancer partly via cyclin D1. J Exp Clin Cancer Res. 2016;35:15. 26791264 10.1186/s13046-016-0294-2 PMC4721151

[26] Perri F , Pezzullo L , Chiofalo MG , et al. Targeted therapy: a new hope for thyroid carcinomas. Crit Rev Oncol Hematol. 2015;94(1):55‐63. 25465739 10.1016/j.critrevonc.2014.10.012

[27] Liu Y , Liu X , Zhang X , Deng J , Zhang J , Xing H . lncRNA DLX6‐AS1 promotes proliferation of laryngeal cancer cells by targeting the miR‐26a/TRPC3 pathway. Cancer Manag Res. 2020;12:2685‐2695. 32368147 10.2147/CMAR.S237181 PMC7183358

[28] Kong WQ , Liang JJ , Du J , Ye ZX , Gao P , Liang YL . Long noncoding RNA DLX6‐AS1 regulates the growth and aggressiveness of colorectal cancer cells via mediating miR‐26a/EZH2 axis. Cancer Biother Radiopharm. 2020. 10.1089/cbr.2020.3589 32379493

[29] Wu SB , Wang HQ . Upregulation of long noncoding RNA DLX6‐AS1 promotes cell growth and metastasis in esophageal squamous cell carcinoma via targeting miR‐577. Eur Rev Med Pharmacol Sci. 2020;24(3):1195‐1201. 32096208 10.26355/eurrev_202002_20171

[30] Liu P , He W , Lu Y , Wang Y . Long non‐coding RNA LINC00152 promotes tumorigenesis via sponging miR‐193b‐3p in osteosarcoma. Oncol Lett. 2019;18(4):3630‐3636. 31579407 10.3892/ol.2019.10700 PMC6757312

[31] Choi KH , Shin CH , Lee WJ , Ji H , Kim HH . Dual‐strand tumor suppressor miR‐193b‐3p and ‐5p inhibit malignant phenotypes of lung cancer by suppressing their common targets. Biosci Rep. 2019;39(7):1‐12. 10.1042/BSR20190634 PMC6630026 31262974

[32] Qian Y , Song W , Wu X , et al. DLX6 antisense RNA 1 modulates glucose metabolism and cell growth in gastric cancer by targeting microRNA‐4290. Dig Dis Sci. 2020;66:460‐473. 32239379 10.1007/s10620-020-06223-4

[33] Wang H , Chen W , Yang P , Zhou J , Wang K , Tao Q . Knockdown of linc00152 inhibits the progression of gastric cancer by regulating microRNA‐193b‐3p/ETS1 axis. Cancer Biol Ther. 2019;20(4):461‐473. 30404587 10.1080/15384047.2018.1529124 PMC6422511

[34] He W , Huang Y , Jiang CC , et al. miR‐100 inhibits cell growth and proliferation by targeting HOXA1 in nasopharyngeal carcinoma. Onco Targets Ther. 2020;13:593‐602. 32021301 10.2147/OTT.S228783 PMC6980857

[35] Li X , Pang L , Yang Z , Liu J , Li W , Wang D . LncRNA HOTAIRM1/HOXA1 axis promotes cell proliferation, migration and invasion in endometrial cancer. Onco Targets Ther. 2019;12:10997‐11015. 31853186 10.2147/OTT.S222334 PMC6917485

