# Supplementary figures and images for: Silencing long non‐coding RNA DLX6‐AS1 or restoring microRNA‐193b‐3p enhances thyroid carcinoma cell autophagy and apoptosis via depressing HOXA1
Source: J Cell Mol Med. 2021 Sep 12;25(19):9319–30. doi: 10.1111/jcmm.16868 (PMC8500975; doi:10.1111/jcmm.16868)

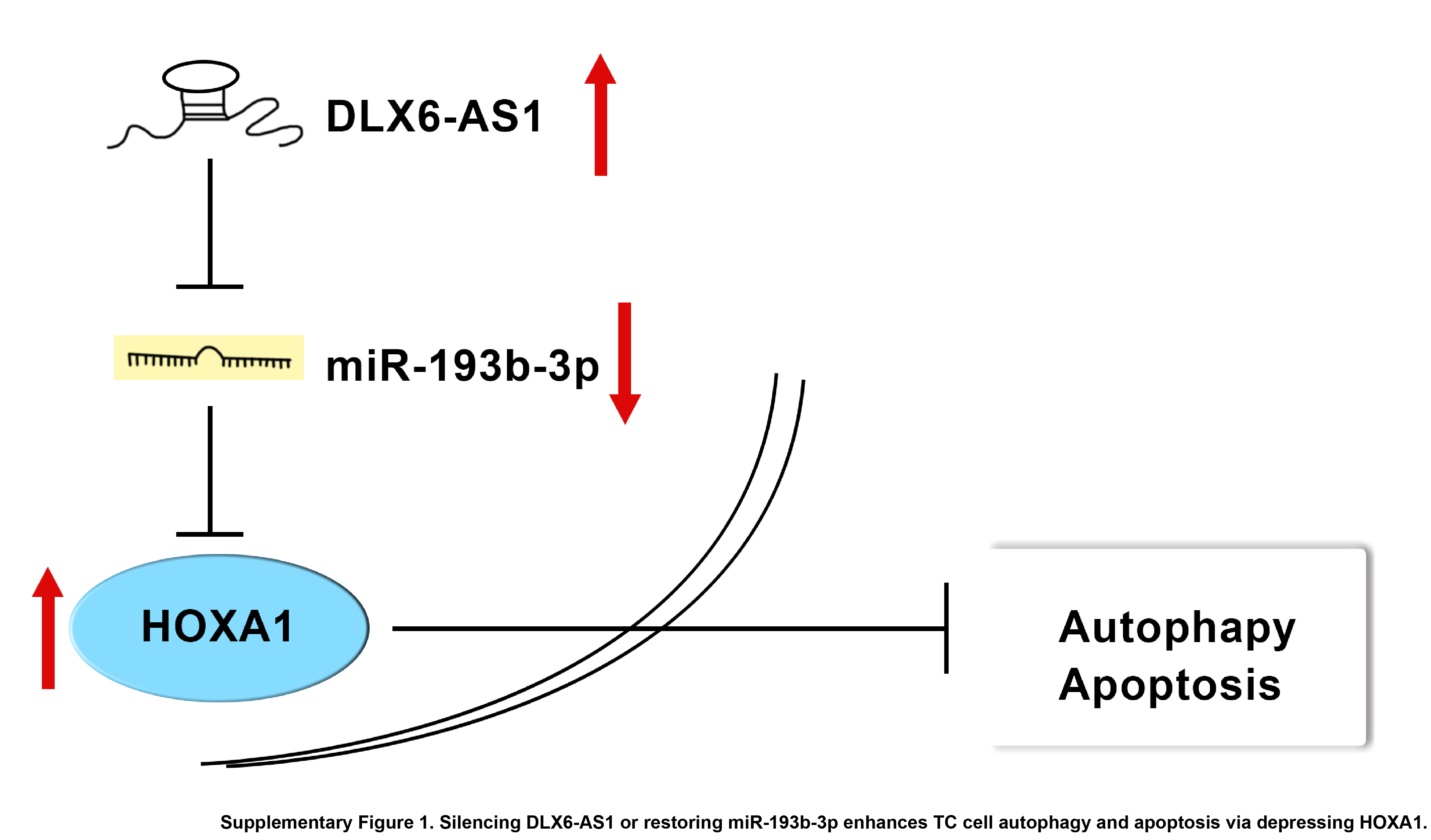

Supplement: Supplementary file 1 — Fig S1 [file JCMM-25-9319-s001.jpg]
